# Assessing the Effects of the Topical Application of L-Menthol on Pain-Related Somatosensory-Evoked Potentials Using Intra-Epidermal Stimulation

**DOI:** 10.3390/brainsci13060918

**Published:** 2023-06-06

**Authors:** Taiki Makibuchi, Koya Yamashiro, Sayaka Anazawa, Tomomi Fujimoto, Genta Ochi, Koyuki Ikarashi, Daisuke Sato

**Affiliations:** 1 Institute for Human Movement and Medical Sciences, Niigata University of Health and Welfare, Niigata 950-3198, Japan; wtm22005@nuhw.ac.jp (T.M.); fujimoto@nuhw.ac.jp (T.F.); ochi@nuhw.ac.jp (G.O.); koyuki-ikarashi@nuhw.ac.jp (K.I.); daisuke@nuhw.ac.jp (D.S.); 2 Field of Health and Sports, Graduate School of Niigata University of Health and Welfare, Niigata 950-3198, Japan; wtm22006@nuhw.ac.jp; 3 Department of Health and Sports, Niigata University of Health and Welfare, Niigata 950-3198, Japan

**Keywords:** L-menthol, pain-related somatosensory-evoked potentials, transient receptor potential melastatin 8, intra-epidermal electrical stimulation

## Abstract

L-menthol is known to activate transient receptor potential melastatin 8 (TRPM8) and induce analgesia to thermal stimuli. However, since thermal stimulation leads to the interaction among the other TRP channels, it was unclear whether L-menthol causes analgesia to stimuli other than thermal stimuli. Therefore, we aimed to investigate whether activating TRPM8 via topical application of 10% menthol solution attenuates pain-related somatosensory-evoked potentials (pSEPs) and affects numerical rating scale (NRS) score using intra-epidermal electrical stimulation (IES). We applied 10% L-menthol or control solution on the dorsum of the right hand of 25 healthy participants. The pSEP and NRS, elicited by IES, and sensory threshold were measured before and after each solution was applied. The results showed that the topical application of 10% L-menthol solution significantly reduced N2–P2 amplitude in pSEPs compared with the control solution. Moreover, the N2 latency was significantly prolonged upon the topical application of L-menthol solution. NRS scores were similar under both conditions. These results suggest that topical application of L-menthol does not alter subjective sensation induced using IES, although it may attenuate afferent signals at free nerve endings even with stimuli that do not directly activate TRP channels.

## 1. Introduction

The International Association for the Study of Pain (IASP) defines pain as “An unpleasant sensory and emotional experience associated with, or resembling that associated with, actual or potential tissue damage [1]”. Physical pain is intrinsically unpleasant and aversive. It drives behaviors that avoid bodily injury when interacting with the environment [2]. Pain that has served its role as a warning signal should be immediately relieved. Thus, transient receptor potential melastatin 8 (TRPM8) channels have been identified as a potential target to induce analgesia [3] and are being actively pursued as targets for drug discovery [4,5].

L-menthol, the natural cooling product of peppermint, has historically been used topically to alleviate various painful conditions [6,7]. In general, menthol selectively activates TRPM8, a nonselective, calcium-permeable channel expressed in a subset of sensory neurons in the dorsal root ganglion [8], specifically the pain- and temperature-sensing neurons [9], which is activated at cold temperatures (8–28 °C) [9,10,11,12,13]. Moreover, this channel is associated with analgesic effects induced by natural cooling products, such as icilin [12,13] and menthol [3,10,11,13,14,15,16]. These natural cooling products are known as TRPM8 “agonists” (in detail in González-Muñiz et al., 2019) [17]. On the other hand, F-05105679, AMG-333, and cannabidivarin are the three TRPM8 antagonists [17]. TRPM8 is present in thinly myelinated Aδ-fibers and unmyelinated C-fibers of sensory nerves and skin cells [6,18,19]. TRPM8 seems to be associated with pain modulation in the peripheral and central nervous systems [12,20]. In the peripheral nerve system, TRPM8 transduces innocuous cool temperatures to trigger cool perception, behavioral thermoregulation, and analgesia; it also transmits nociceptive signals [21]. It is suggested that its interactions with different concentrations of menthol and chemical or physical stimuli and the structure of the TRPM8 channel determine the pain status [22]. At the level of the spinal cord in the central nerve system, Group II/III metabotropic glutamate receptors could centrally mediate analgesia induced using menthol, icilin, and cooling [12]. These receptors can inhibit nociceptive responses [23,24,25], and they are located pre- and post-synaptically at primary afferent synapses [12,23,26]. Therefore, the analgesic effect induced by L-menthol might be associated with TRPM8 and glutamate receptors at the spinal cord. The concentration of menthol in various over-the-counter topical products for pain relief ranges from 5% to 16% [22,27]. A previous study showed that the cutaneous application of 10% menthol solution to the forehead and temporal areas of the affected side of patients with migraine was a significantly effective placebo for pain relief [28]. L-menthol was found to induce TRPM8-dependent acute and inflammatory pain relief using various concentrations and administration routes [7]. Although low-concentration L-menthol solution has analgesic effects, topical application of its high-concentration solution induces cold hypersensitivity [12,18,19,29,30]. In fact, topical application of 40% L-menthol was found to induce cold pain and hyperalgesia in a human study [31]. Therefore, it can be inferred that the topical application of L-menthol is helpful for pain relief in both humans and animals; however, there is still a paucity of research on the analgesic mechanism in humans.

The pain-related somatosensory-evoked potentials (pSEPs) greatly help in evaluating response to noxious stimulation. These pSEPs have a distinct subsequent negative peak (N2) and a late central positive peak (P2) [32]. Previous studies suggested that the topical application of L-menthol reduced the N2–P2 amplitude, and longer latency was induced by noxious thermal stimulation, i.e., laser and contact heat and cold stimuli [10,33]. Furthermore, the topical application of L-menthol was shown to decrease the subjective intensity of pain, as assessed using the visual analog scale (VAS) induced by thermal stimulation [10]. Additionally, the use of topical menthol-based analgesics decreased the level of perceived discomfort caused due to the delayed onset of muscle soreness [34]. So, L-menthol exhibited inhibitory effects on simultaneously established pain, hypersensitivity, and neurogenic inflammation [6]. In addition, animal studies show that TRPM8 was essential for L-menthol to inhibit nocifensive responses triggered by the TRPV1- and TRPA1-activated pain pathways [7].

TRP channels are thermosensitive ion channels. Interactions between TRP channel agonists (such as menthol, capsaicin, and cinnamaldehyde) and noxious and innocuous thermal stimuli (contact heat stimulation, laser stimulation, and cold stimulation) have been studied [10,33]. However, previous studies have not been able to evaluate the effects of menthol alone purely because the warming/cooling stimulus itself activates TRP channels except for TRPM8. Therefore, we used intraepidermal electrical stimulation (IES) to activate Aδ-fibers [32] without changing the temperature to eliminate the effect of the stimulus-induced activation of other TRP channels. In this study, if the analgesic effects of L-menthol with IES are demonstrated, treatment to activate TRPM8 would be clinically helpful. Therefore, we examined whether activating TRPM8 with a topical 10% menthol solution alters pain-related evoked potentials and NRS using IES. We hypothesized that applying 10% menthol solution would attenuate the induced potentials and NRS and prolong the latency.

## 2. Materials and Methods

### 2.1. Participants

Twenty-five healthy adults were recruited (thirteen males and twelve females). After fully explaining the study’s objectives and methodology, each participant was given written information on the experiment. This sample size was larger than the minimum of 24 that was needed for 80% power and a significance level of 5% based on an effect size of 0.60. The mean age of the participants was 20.4 ± 1.2 years, their mean height was 166.8 ± 7.7 cm, and their mean weight was 58.8 ± 8.3 kg. Exclusion criteria had a neurologic, metabolic, or psychiatric condition, suffering from chronic pain or being in pain, having a history of allergic reaction to mint, and suffering from a dermatologic condition. The study was conducted as per the principles of the Declaration of Helsinki and approved by the ethics committee of Niigata University of Health and Welfare, Niigata, Japan (approval number 18913). 

### 2.2. Intraepidermal Electrical Stimulation

IES was used for noxious stimulation developed by Inui et al. (2002) [32]. This stimulation can selectively activate cutaneous Aδ-fibers. In this study, stainless steel concentric bipolar needle electrodes (Nihon Kohden, Tokyo, Japan NM-980W) were used for the IES. The anode was an outer ring 1.2 mm in diameter, and the cathode was an inner needle that was 0.2 mm in length. Three electrodes were used at 10 mm intervals [35]. Pressing the electrode against the dorsum of the right hand, the needle tip was inserted in the epidermis and superficial part of the dermis where nociceptors were located. Inter-stimulus intervals were set as 20 ms due to enhanced brain responses. The electrical stimulation was a triangular wave pulse [32,36]. Sensory thresholds were measured with an intensity of 0.01 mA, and the current was increased in steps of 0.01 mA [37]. The threshold was determined when the participants felt a pricking sensation. Usually, the pricking sensation disappeared after stimulus intensity decreased; however, some participants experienced a weaker, similar sensation. 

### 2.3. Agonist and Control Solutions

A 10% L-menthol solution was made by diluting menthol (>98% L-menthol; 134-03751; FUJIFILM Wako Pure Chemical Corporation, Osaka, Japan) with dimethyl sulfoxide (>98% DMSO; 13406-55; NACALAI TESQUE, INC., Kyoto, Japan) and propylene glycol (>98% propylene glycol; 29217-45; NACALAI TESQUE, INC., Kyoto, Japan) at concentrations of 2% and 98%, respectively. The corresponding control solution was prepared using only DMSO and PG at concentrations of 2% and 98%, respectively. This control solution allowed us to exclude the effect of components other than menthol when a significant difference was found in the menthol solution and not in the control, thereby enabling us to assess the effect of L-menthol. In this study, we used DMSO to dissolve L-menthol crystal. The 2% DMSO did not independently alter cutaneous vascular function [38]. 

### 2.4. EEG Recording

A SynAmps amplifier system and scan 4.3 software (Neuroscan, El Paso, TX, USA) were used for EEG acquisition. Electrode impedance was maintained below 5 kΩ. The EEG was recorded using five scalp electrodes placed at Fz, Cz, Pz, C3, and C4 according to the 10–20 system. The left earlobe was used as a reference. EEG signals were recorded with a notch filter (50 Hz) at a sampling rate of 1000 Hz. According to our previous studies, trials with responses exceeding ±100 μV were excluded from averaging [39,40,41]. The offline band-pass filter was set at 0.5–30 Hz. Twenty trials were averaged for each participant. Responses were analyzed from 100 ms before the stimulus onset (baseline) to 600 ms after the stimulus onset.

### 2.5. Experimental Procedures

**Figure 1:**
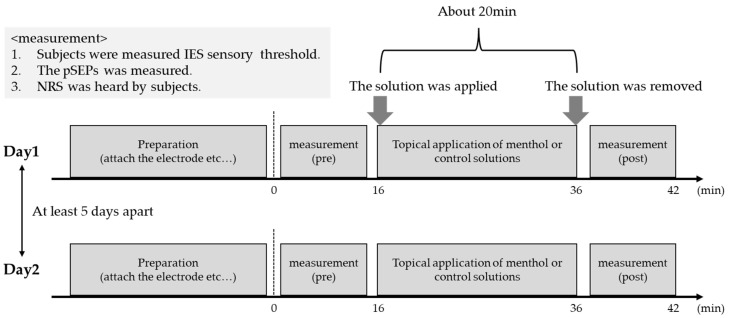
The experimental procedure.

The experimental procedures are shown in Figure 1. First, the measurements (pre) were performed before the topical application of menthol or control solution. To avoid habituation and fatigue, IES was given 20 times at the dorsum of the right hand based on a previous study, wherein stimulation was given 10–20 times [37,42,43], and the inter-trial interval was 12 s. After the end of the stimulus, subjects were asked to rate, using the numerical rating scale (NRS), how they felt when given the IES. The NRS consists of integers from 0 to 100, which correspond to “no pain” and “the worst possible pain”, respectively. Using the previously described method, L-menthol or control solution was then topically administered to the stimulation area and allowed to soak for 20 min [10]. Each solution was applied using a brush until the stimulation area was covered. These solutions were applied to all participants in random order. Moreover, the two applications of each solution were spaced at least 5 days apart to avoid residual the effect of the topical application of another solution. In previous study, the analgesic cream containing 6% L-menthol was effective for 1 h [44]. Finally, the same measurements (post) were performed as before the topical application of menthol or control solution.

### 2.6. Analyses

In line with a previous study [45], we performed pSEPs component analyses using only the Cz electrode. The stimulation that elicited the negative peak was defined as the most negative deflection occurring between 170 ms and 250 ms following the stimulus onset. In contrast, the positive peak was defined as the most positive deflection occurring between 250 ms and 350 ms following the stimulus onset, according to a previous study [46]. The peak-to-peak amplitudes (N2–P2), peak (N2 and P2) latency, sensory threshold, and NRS were calculated. All statistical analyses were conducted using GraphPad Prism v 9.1.0 (San Diego, CA, USA). The Shapiro-Wilk test was performed to confirm the normally distributed before the paired-sample t-test. If the models were not found to be normally distributed, we performed the Wilcoxon signed-rank test. The threshold for statistical significance was set at *p* < 0.05 for all tests. Results are expressed as median [interquartile range, IQR].

## 3. Results

### 3.1. Sensory Threshold

There were no significant differences in the sensory threshold for IES before the topical application of L-menthol and control solutions (0.06 [0.04–0.10] mA vs. 0.07 [0.05–0.09] mA, *p* = 0.84).

### 3.2. Effect of L-Menthol Application

The perception intensity evaluated by NRS did not differ significantly between L-menthol application (20.0 [13.0–40.0] vs. 25.0 [15.0–47.5], *p* = 0.55) and the control (20.0 [10.0–40.0] vs. 25.0 [14.0–42.5], *p* = 0.64). The IES electrical threshold did not differ significantly between L-menthol (0.06 [0.04–0.10] vs. 0.07 [0.05–0.10], *p* = 0.47) and the control (0.07 [0.05–0.09] vs. 0.06 [0.05–0.09], *p* = 0.83). The topical application of 10% menthol elicited either a cool sensation or no sensation, sometimes with elements of cold or warmness.

### 3.3. Effect of L-Menthol Application on pSEPs

**Figure 2:**
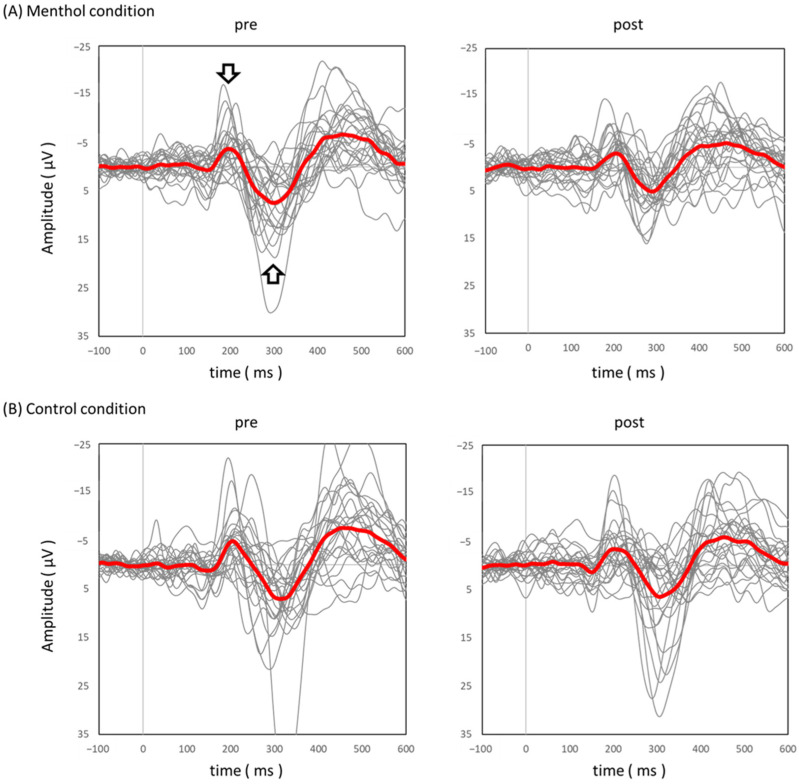
Grand averaged pSEP. Grand averaged waveforms recoded at the vertex electrode (Cz) following IES in menthol condition (**A**) and in control condition (**B**). The red and gray lines show averaged and each subject’s pSEPs wave, respectively. The negative and positive peaks indicated using arrows represent N2 and P2, respectively.

**Figure 3:**
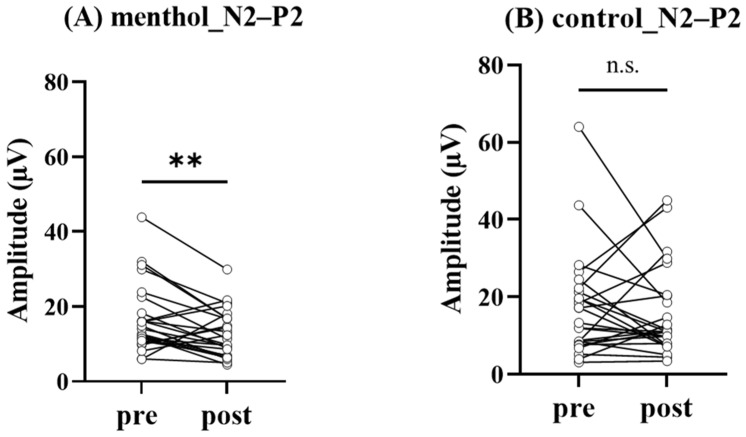
The comparison of N2–P2 amplitude observed before and after the topical application of each solution. The N2–P2 amplitude in L-menthol condition (**A**) and in control condition (**B**). ** *p* < 0.01.

**Figure 4:**
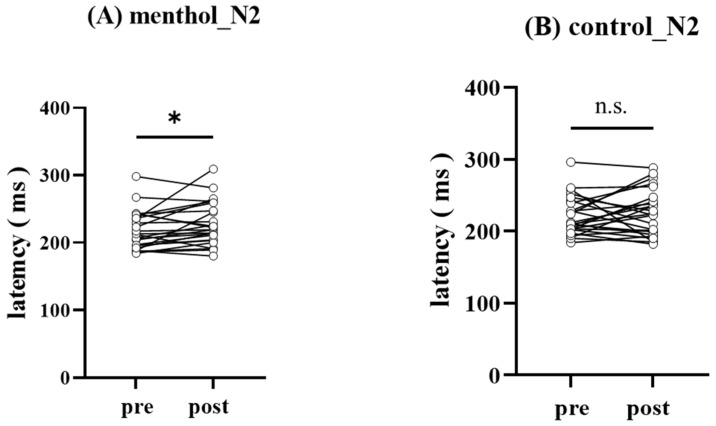
The comparison of N2 latency observed before and after the topical application each solution. The N2 latency in menthol condition (**A**) and in control condition (**B**). * *p* < 0.05.

Figure 2 showed grand-averaged pSEPs in menthol (A) and control (B) conditions. The N2–P2 amplitude on the Cz electrode was significantly decreased under menthol conditions (14.0 [10.9–20.4] µV vs. 11.4 [6.9–16.8] µV, *p* = 0.007; Figure 3). In the control condition, there was no significant difference in N2–P2 amplitude on the Cz electrode (13.3 [8.1–21.70] µV vs. 11.1 [7.7–20.4] µV, *p* = 0.63; Figure 3). As a result, N2 latency (212.0 [194.0–238.5] ms vs. 219.0 [203.5–253.0] ms, *p* = 0.04; Figure 4) was significantly prolonged under the menthol condition. No significant differences were found in P2 latency (306.0 [272.0–333.5] ms vs. 291.0 [274.0–325.5] ms, *p* = 0.45) under the menthol condition.

## 4. Discussion

A previous study demonstrated TRPM8-mediated L-menthol analgesia using noxious heat/cold stimulation, a thermal stimulus that activates TPR channels [10]. The L-menthol analgesic effect has been confirmed in humans [10,28] and animals [7]. Therefore, we investigated L-menthol’s analgesic effect using IES in the present study, excluding the stimulus-induced TRP channel activation effects. As a result, there was no significant difference in NRS from the subjective pain rating. However, our results showed that the N2–P2 amplitude in pSEPs was significantly reduced by the topical application of the 10% L-menthol solution. In addition, N2 latency was significantly prolonged by the topical application of the 10% L-menthol solution. Therefore, the activation of TRPM8 could attenuate afferent signals of free nerve endings but not change the level of subjective pain.

There are two possible reasons for the absence of a difference in NRS. First, it could be that the intensity of stimulation by the IES was not high. In the present study, the NRS was 20 on a scale of 0 to 100. This score was consistent with the findings of a previous study that reported a VAS of 2–6 (0–10) [35]. Moreover, the latency of N2 and P2 was within the range of latency reported in a previous study [46]. It suggested that Aδ-fibers were activated by IES in this study. However, the low value of NRS in both pre-conditions (control and L-menthol) may have prevented a statistically significant difference even if analgesia had occurred. In fact, previous studies reported a higher NRS score against noxious stimulus before the topical application of L-menthol solution than that observed in the present study [10]. Second, the concentration of L-menthol may have been another reason. In a previous study conducted using a similar protocol with L-menthol, analgesia was produced using a 20% concentration of menthol [10]. This result suggests that a 10% concentration would not be sufficient to have an effect leading to analgesia. The 20% concentration was not used in this study because of the possibility of ensuing inflammation. We also considered that L-menthol at a 10% concentration is within the range of clinically used analgesics [22]. However, this study only found that afferent signals elicited by IES were attenuated via the topical application of a 10% L-menthol solution. The subjective pain sensations evaluated using NRS may not have been changed by the intensity of weak nociceptive electrical stimulation as mentioned above.

In the present study, the N2–P2 amplitude was decreased after the topical application of L-menthol. Pain is said to involve not only sensory information but also emotional processing [1,2]. The pSEPs N2–P2 components are said to be involved in sensory discrimination and cognitive factors [36]. L-menthol produces analgesia to other stimuli by activating TRPM8 [22]. In the present study, the brain responses showed a decrease in amplitude; however, the mechanism by which this occurred is not clear. A previous study showed that N2–P2 is thought to reflect the middle of the scalp activity, such as primary and secondary somatosensory, anterior cingulate cortex, insula, prefrontal cortex, and supplementary motor areas [36,47]. The analgesic effect of topical L-menthol was a decrease in the N2–P2 amplitude, which is consistent with the findings of a previous study [10]. A previous study reported that the topical application of menthol induced inhibiting primary afferent signals at the Aδ-fibers [10]. These inhibiting afferent signals may alter pSEPs elicited by IES.

A previous study showed that increasing the baseline temperature of contact heat evoked potentials, nociceptive stimuli, significantly less N2 jitter, and larger N2 and P2 amplitudes [48]. In addition, the use of relatively high energies for stimulation probably yielded a higher degree of neuronal synchronization and, therefore, could account for more prominent responses [49]. Thus, the topical application of L-menthol may induce the delay of N2 latency and reduce the N2–P2 amplitude to increase the stimulus jitter in this study, similarly to the previous study [10].

Our results support the notion that topical application of L-menthol selectively activates TRPM8 in Aδ-fiber terminals to induce analgesia [22]. TRP channels can be activated via various stimuli, such as thermal, chemical, and mechanical stimuli [50]. The interaction between L-menthol and noxious thermal stimulation was an issue in a previous study. In this study, this interaction was almost eliminated using IES, which selectively activated the free nerve ending of the Aδ-fibers [32,35]. 

## 5. Limitation of This Study

L-menthol has a mint-like fragrance [22,51]. In this study, 10% L-menthol solution was prepared in DMSO and PG; however, the mint-like fragrance was noticeable when the solution was topically applied, and it is a major limitation of this study. As it is possible that participants were aware of the solution applied on them (either control or L-menthol solution), the placebo effect could not be avoided. However, owing to the possibility of experiencing a paradoxical burning sensation upon the topical application of L-menthol solutions on mucosal tissues such as the eyes and nose, participants were instructed that the hand on which the solution was applied should be kept away from their faces. In addition, the precautions that were to be taken while applying the menthol and control solutions were explained to the subjects, but the fact that the solution had analgesic effects was not disclosed while explaining the experiment. Interestingly, NRS did not change, but N2–P2 amplitude and latency changed, which suggests the afferent nerve signal attenuation. Therefore, it is considered that both placebo and nocebo effects were minimal. Future studies should be performed using the control and menthol solutions while considering the effect of smell, as has been demonstrated in a previous study [30].

## 6. Conclusions

We found that topical application of L-menthol can reduce N2–P2 amplitude and increase N2 latency to IES, which might be caused by attenuated afferent signals at least [10]; however, no change in the subjective pain was observed. Therefore, topical application of L-menthol might attenuate only afferent signals induced by IES stimulation that does not activate TRP channels.

## Data Availability

The data presented in this study are available on request from the corresponding author.
